# A Retrospective Cohort Study From the National Inpatient Sample Database (2016-2019): Does Obesity Affect the Outcomes of Hospitalization Due to Hepatocellular Carcinoma?

**DOI:** 10.7759/cureus.62352

**Published:** 2024-06-14

**Authors:** Sagar Pandey, Madhav Changela, Kapilkumar Manvar, Amulya Bellamkonda, Aditya Keerthi Rayapureddy, Binit Aryal, Kalendra Kunwar, Samaj Adhikari, Dhruvanshu Patel, Kalpana Panigrahi, Madhumati Kalavar

**Affiliations:** 1 Internal Medicine, One Brooklyn Health/Interfaith Medical Center, Brooklyn, USA; 2 Hematology and Medical Oncology, One Brooklyn Health/Brookdale University Hospital Medical Center, Brooklyn, USA; 3 Internal Medicine, Decatur Memorial Hospital, Decatur, USA; 4 Internal Medicine, St. Luke's University Health Network, Bethlehem, USA; 5 Hematology and Medical Oncology, One Brooklyn Health/Interfaith Medical Center, Brooklyn, USA

**Keywords:** cohort, mortality, outcomes, hepatocellular carcinoma, obesity

## Abstract

Introduction: Obesity is commonly reported to be associated with hepatocellular carcinoma (HCC) along with higher risks of mortality. However, there is a significant research gap regarding the outcomes of hospitalization due to HCC among obese patients compared to those without obesity. This study compares the outcomes of hospitalization among those two groups.

Methods: A total of 50,845 patients admitted from 2016 to 2019 with a principal admission diagnosis of HCC were identified using the International Classification of Disease 10 (ICD-10) coding from the National Inpatient Sample (NIS) database. Patients with a body mass index (BMI) >30 were stratified into the obese cohort, and those with BMI ≤30 into the non-obese cohort as per the ICD-10 coding criteria for obesity based on BMI. The primary outcome of the study was mortality, whereas the length of stay, total hospitalization charges, acute kidney injury (AKI), sepsis, and shock were the secondary outcomes. We also compared additional complications such as ascites, portal hypertension, acute liver failure, disseminated intravascular coagulation (DIC), hepatic encephalopathy, and hepatorenal syndrome between the two groups. A multivariate regression model was used to estimate the effect of obesity on outcomes of hospitalization due to HCC.

Results: The obese cohort comprised 10.64% of the study population, whereas the non-obese cohort comprised 89.36% of the study population. Compared to the non-obese cohort, the obese cohort of patients with HCC were more likely to have a higher comorbidity index (CCI ≥4: 79.76% in the obese vs 71.17 % in the non-obese cohort). Obesity was found to be a protective factor for in-hospital mortality; that is, the odds of in-hospital mortality among the obese cohort was 0.713 times than that of the non-obese group of patients with HCC. The obese cohort had a higher mean length of stay (6.3 days vs 5.6 days; p value: <0.001) and total hospitalization charges (109,108$ vs 85,406$; p value: <0.001), which was further validated on multivariate analysis. The obese cohort had 1.26 times odds of developing AKI compared to the non-obese cohort (p value: 0.005). Sepsis, shock, and other complications such as acute liver failure, DIC, hepatic encephalopathy, hepatorenal syndrome, and portal hypertension were not significantly different between the two groups.

Conclusion: Obesity was associated with reduced in-hospital mortality among patients with HCC. However, obese patients with HCC were found to have higher healthcare resource utilization in terms of length of stay and total hospitalization charge along with the development of AKI. Clinicians should be mindful of the potential longer length of stay and associated complications such as AKI while managing obese patients with HCC. Contrary to commonly held notions, obesity and its relation with in-hospital mortality reported in this study warrants further explorative research.

## Introduction

The National Health and Nutrition Examination Survey 2017-2018 has reported that an estimated 42.4% of US adults aged ≥20 years were living with obesity, which included 9% of those with severe obesity. Furthermore, another 30.7% were reported to be overweight. Overweight, obesity, and severe obesity were categorized with respective mass index (BMI) of 25.0-29.9, ≥30.0, and ≥ 40.0. This translates to an approximately one in three adults being overweight, more than two in five adults having obesity, and one in eleven adults having severe obesity. The prevalence of obesity and severe obesity among US adults has shown an approximate 12 percentage points increase in the year 2017-2018 compared to that in 1999-2000 [1]. Although obesity has been widely linked to a wide range of cardiovascular morbidities [2], studies have also reported its association with organ-specific cancer mortalities. Specifically, obese or overweight subjects were found to have an elevated risk of mortality from rectum, bladder, colon, and liver cancer along with lymphoma in men [3].

Globally, an estimated 826,000 cases of liver cancer were reported in 2018 with 80% of the burden contributed by hepatocellular carcinoma (HCC), 14.9% by intrahepatic cholangiocarcinoma, and the rest constituting other specified histology [4]. With regard to the etiological burden of liver cancer globally, an epidemiological shift has been reported behind the incidence of liver cancer from viral etiologies (hepatitis B and C virus to non-viral) to non-viral metabolic entities such as alcohol-related and metabolic dysregulation such as hepatic steatosis. This is supported by the fact that non-alcoholic steatohepatitis was ranked fifth among etiological factors behind the development of liver cancer in 1990, contributing 4.7% to the global burden of liver cancer. This is in contrast to the epidemiological findings in 2019 where non-alcoholic steatohepatitis has been reported to contribute 6.8% to the global burden of liver cancer and ranks fourth among the commonest causes of liver cancer following hepatitis B, hepatitis C, and alcohol use [5]. The risk of liver cancer with BMI was demonstrated in a meta-analysis of 21 prospective studies involving 17,624 cases of primary liver cancer. In the study, a five-unit increase in BMI was associated with a 39% increased risk of primary liver cancer. This summary relative risk with a five-point increase in BMI, and risk of primary liver cancer was positive on subgroup meta-analyses by sex, geographical location, ascertainment of exposure and outcomes, number of cases, duration of follow-up, sample source, and confounders such as alcohol consumption, smoking, hepatitis B, hepatitis C, etc. Compared to the general population with excess weight, cirrhotic individuals along with hepatitis C virus (HCV)-positive patients with excess weight had 59% and 98% increased risk of primary liver cancer, respectively. The risk of primary liver cancer and BMI was seen as most pronounced with a BMI of more than 32 kg/m^2^ [6].

Obese patients with HCC have also been reported to have a higher risk of HCC-related mortality compared to patients with normal BMI. More precisely, the risk of HCC-related mortality was 84% higher in obese male patients and 26% higher in obese female patients with HCC. In contrast, such increased risk was not observed with HCC patients with BMI in the overweight category. To note, the studies included in the meta-analysis did not adjust for the etiology and severity of cirrhosis, HCC cancer stage, and treatment [7]. However, there remains a significant research gap with regard to the impact of obesity on outcomes of hospitalization in patients admitted with a principal diagnosis of HCC. This study explores the effect of obesity on outcomes of hospitalization in terms of mortality and length of stay along with selective hepatic and non-hepatic complications in patients admitted with a principal diagnosis of HCC.

## Materials and methods

Study design and database description

We conducted a retrospective cohort study with a study population comprising adult patients hospitalized with HCC in the United States from the year 2016 to 2019. Patient data were obtained from the Nationwide Inpatient Sample (NIS) database, which is a part of databases developed for the Healthcare Cost and Utilization Project (HCUP). The NIS is the largest publicly available all-payer inpatient healthcare database, which estimates US regional and national inpatient utilization, access, cost, quality, and outcomes from all nonfederal acute care hospitals nationwide. The NIS database approximates a 20% stratified sample of all discharges from US community hospitals, excluding rehabilitation and long-term acute care hospitals. The NIS database contains anonymized clinical and resource-use information that is included in a typical discharge with safeguards to protect the privacy of patients, physicians, and hospitals. Unweighted, the NIS database contains data from around seven million hospital stays and around 35 million weighted estimates of hospitalizations nationwide. Clinical and non-clinical information obtained from the NIS includes the International Classification of Diseases, tenth revision; clinical modification/procedure coding system (ICD-10-CM/PCM) diagnosis, procedure, and external causes of morbidity codes from 2016 to 2019; and anonymized patient demographic characteristics (such as age, sex, race, median household income for zip code) and hospital characteristics (such as location, bed size, teaching status), expected payment source, length of stay, severity and comorbidity measures [8,9].

Ethical statement

The study was conducted in compliance with the set of ethical principles of the Declaration of Helsinki. The NIS database contains anonymized clinical and resource-use information with safeguards to protect the privacy of patients, physicians, and hospitals. As patient data were de-identified, institutional review board (IRB) approval and written consent were not required before proceeding with the study.

Study population

Patients aged 18 years or more, admitted from 2016 to 2019 with a principal admission diagnosis of HCC, were identified using the ICD-10-CM coding system from the NIS database. Patients aged less than 18 years were excluded from the study. ICD-10 code C22.0 was used to query the NIS database 2016-2019 to identify patients admitted with a principal diagnosis of HCC. The study population thus obtained was then stratified into obese and non-obese groups based on the ICD-10-CM coding criteria for obesity based on BMI. Patients with a BMI of more than 30 were stratified into the obese cohort and those with a BMI of 30 or less into the non-obese cohort. ICD-10 code E66.XX was used to identify patients with obesity, and the rest of the study population was segregated into the non-obese cohort. A total of 50,845 hospitalizations with principal admission diagnoses of HCC were included in the study.

Study variables and outcomes

Study variables include the patient's demographic variables, hospital characteristics, and the Charlson Comorbidity Index (CCI). Information about age, sex, race, insurance provider, and median income in the patient’s zip code was collected as the patient’s demographic variables. Hospital characteristics included hospital region, teaching status, bed size, and location. The patient’s comorbid status was categorized using Deyo adaption of CCI for research relying on ICD diagnosis and procedure codes. Deyo adaption of CCI consists of a total of 19 categories of clinical comorbid conditions, which are assigned prespecified scores. CCI predicts the 10-year mortality of the patient based on the CCI scores [10].

The primary outcome of the study was mortality where secondary outcomes included length of stay, total hospitalization charges, acute kidney injury (AKI), sepsis, shock along with hepatic complications such as development of ascites, hepatorenal syndrome, hepatic encephalopathy, portal hypertension, acute liver failure, and disseminated intravascular coagulation (DIC). Mortality, length of stay, and total hospitalization charges were directly coded within the NIS for each hospitalization. The rest of the secondary outcomes were coded using appropriate ICD-10 codes (available in appendices). Potential confounders addressed in the study included age, sex, race, CCI, hospital bed size, hospital region, hospital teaching status, presence of hepatitis B and hepatitis C, and alcohol abuse.

Statistical analysis

Stata® _Version 18 software (StataCorp, Texas) was used for data analysis. All analyses were conducted using the weighted samples for national estimates in adjunct with HCUP regulations for using the NIS database. HCUP rules and regulations were thoroughly followed in utilizing the NIS database. Continuous variables were expressed as mean ± standard deviation and were assessed by Student’s t-test, whereas categorical variables were expressed as frequencies and percentages and assessed by the Pearson chi-square method. To identify all possible confounders, we performed a thorough review of existing literature on obesity and HCC hospitalization, obtained expert opinions from experts in the respective fields, and performed a univariate screening. Following the univariate screening, all the variables with p value less than 0.2 were included in the multivariate regression model to adjust for confounders while calculating the primary and secondary outcomes. Outcomes that were adjusted for included age, sex, race, CCI, hospital bed size, hospital region, hospital teaching status, and comorbidities not accounted for in the CCI such as the presence of hepatitis B and hepatitis C and alcohol abuse. Multivariate linear regression analysis was used for continuous variables, whereas multivariate logistic regression analysis was used for binary/dichotomous variables to compare the outcomes among different groups. All p-values <0.05 were considered statistically significant.

## Results

Demographic characteristics

Out of a total of 142,420,378 hospitalizations from 2016 to 2019, a total of 50,845 patients hospitalized with a principal diagnosis of HCC were included in the study. The obese cohort comprised 10.64% (n=5,410) of the study population, whereas the non-obese cohort comprised 89.36% (n=45,435) of the study population. In terms of gender distribution, male patients were more likely to be non-obese (obese: 67.81% (n=3,669) vs non-obese: 75.76% (n=34,422)), whereas female patients were more likely to be obese (obese: 32.19% (n=1,741) vs non-obese: 24.24% (n=11,013)). Compared to the non-obese cohort, an obese cohort of patients with HCC were more likely to have a higher comorbidity index (CCI ≥4: 79.76% (n=4,315) in obese vs 71.17 % (n=32,336) in the non-obese cohort). Numerically small but statistically significant differences between the obese and non-obese cohorts of patients were seen with regard to insurance provider, median income in patient’s zip code, hospital teaching status, and hospital location. Table 1 demonstrates the baseline patient and hospital characteristics.

**Table 1:** Sociodemographic and clinical characteristics of the study participants $: US dollar

Variable	Obesity, n=5,410 (10.64%)	No Obesity, n=45,435 (89.36%)	p value
Sex, n (%)			
Male	3,669 (67.81)	34,422 (75.76)	<0.001
Female	1,741 (32.19)	11,013 (24.24)	
Mean age in years	64.40	64.80	0.255
Insurance provider, n (%)			
Medicare	2,879 (53.21)	23,313 (51.31)	<0.001
Medicaid	772 (14.27)	9,314 (20.50)	
Private	1,646 (30.43)	10,877 (23.94)	
Uninsured	113 (2.08)	1,931 (4.25)	
Charlson comorbidity index, n (%)			
0-3	1,095 (20.24)	13,099 (28.83)	<0.001
³4	4,315 (79.76)	32,336 (71.17)	
Median income in patient zip code, n (%)			
$1 – 51,999	1,508 (27.87)	15,121 (33.28)	0.0014
$52,000 - 65,999	1,406 (25.99)	11,218 (24.69)	
$66,000 - 87,999	1,427 (26.37)	10,041 (22.10)	
$88,000+	1,070 (19.77)	9,055 (19.93)	
Hospital region, n (%)			
Northwest	1,015 (18.76)	9,891 (21.77)	0.0012
Midwest	1,130 (20.89)	7,351 (16.18)	
South	1,955 (36.14)	17,679 (38.91)	
West	1,310 (24.21)	10,514 (23.14)	
Hospital teaching status, n (%)			
Non-teaching	565 (10.44)	7,197 (15.84)	< 0.001
Teaching	4,845 (89.56)	38,238 (84.16)	
Hospital bed size, n (%)			
Small	570 (10.54)	5,475 (12.05)	0.2029
Medium	1,190 (22.0)	10,564 (23.25)	
Large	3,650 (67.47)	29,533 (65)	
Race, n (%)			
White	3,343 (61.80)	23,208 (51.08)	<0.001
Black	664 (12.28)	8,065 (17.75)	
Hispanic	997 (18.43)	7,097 (15.62)	
Asian or Pacific Islander	156 (2.88)	4,521 (9.95)	
Native American	47 (0.86)	354 (0.78)	
Other	202 (3.74)	2,190 (4.82)	
Hospital location, n (%)			
Rural	80 (1.48)	1,263 (2.78)	0.0108
Urban	5,330 (98.52)	44,172 (97.22)	
Hepatitis B	200 (3.7)	4,316 (9.5)	<0.0001
Hepatitis C	1,500 (27.73)	18,960 (41.73)	<0.0001
Alcoholism	645 (11.92)	7,088 (15.60)	0.0021

Primary outcome

The incidence of in-hospital mortality was higher among non-obese patients with HCC (5.64% (n=305) in the obese cohort vs 8.41% (n=3821) among the non-obese cohort; p value: 0.0021). This relationship between in-hospital mortality among obese and non-obese patients with HCC was true following adjustment of potential confounding factors too; that is, the odds of in-hospital mortality among the obese cohort was 0.713 times that with a non-obese group of patients with HCC.

Secondary outcomes

The obese cohort of patients with HCC had significantly higher measures of healthcare utilization assessed in terms of length of stay and total hospitalization charges. The mean length of stay of the obese cohort was significantly higher compared to that of the non-obese cohort (6.3 days vs 5.6 days; p value <0.001), which was further validated in multivariate analysis (adjusted coefficient: 0.74 with p value <0.001). A similar association was seen with total hospitalization charges where the obese group had higher hospitalization costs compared to the non-obese group (109,108$ vs 85,406$; p value <0.001). The association held true in multivariate analysis (adjusted coefficient: 20,458; p value <0.001). Similarly, the incidence of AKI (obese group: 27.63% (n=1,495) vs the non-obese group: 22.67% (n=10,300); p value: 0.002) and portal hypertension (obese group: 24.86% (n=1,345) vs the non-obese group: 21.28% (n=9,669); p value: 0.0092) was higher in the obese group compared to the non-obese group. After adjusting for confounding variables, the obese cohort had 1.26 times the odds of developing AKI compared to the non-obese cohort (p value: 0.005). However, the odds of developing portal HTN were similar among the obese and non-obese groups after adjusting for confounding variables (OR: 1.12 with p value: 0.2). On the other hand, development of ascites was seen higher among the non-obese cohort (obese group: 30.41% (n=1,645), non-obese group: 36.54% (n=16,602); p value <0.001). This was further validated on multivariate analysis after adjusting for confounding variables (OR: 0.72 with p value <0.001). No significant differences were seen among obese and non-obese cohorts of patients in terms of development of shock, sepsis, acute liver failure, DIC, hepatic encephalopathy, or hepatorenal syndrome. Table 2 shows the results of the effect of obesity on outcomes of hospitalization due to HCC after adjusting for confounders on multivariate regression analysis.

**Table 2:** Multivariate regression analysis on the effect of obesity on the outcomes of hospitalization due to hepatocellular carcinoma OR: odds ratio, CI: confidence interval, LOS: length of stay, AKI: acute kidney injury, DIC: disseminated intravascular coagulation, HTN: hypertension, $: US dollar

Outcomes	Obesity n=5,410 (10.64%)	No obesity n=45,435 (89.36%)	p value
Mortality, n (%)	305 (5.64)	3,821 (8.41)	0.0021
Adjusted OR (95% CI)	0.713 (0.54-0.94)		0.018
Unadjusted OR (95% CI)	0.65 (0.49-0.85)		0.002
LOS (days)	6.3	5.6	<0.001
Adjusted coefficient (95% CI)	0.74 (0.36-1.11)		<0.001
Unadjusted coefficient (95% CI)	0.70 (0.33-1.06)		<0.001
AKI, n (%)	1,495 (27.63)	10,300 (22.67)	0.002
Adjusted OR (95% CI)	1.26 (1.07-1.47)		0.005
Unadjusted OR (95% CI)	1.3 (1.13-1.49)		0.000
Shock, n (%)	165 (3.05)	1,227 (2.7)	0.498
Adjusted OR (95% CI)	1.11 (0.77-1.61)		0.554
Unadjusted OR (95% CI)	1.135 (0.78-1.63)		0.498
Sepsis, n (%)	60 (1.11)	691 (1.52)	0.2861
Adjusted OR (95% CI)	0.73 (0.39-1.34)		0.309
Unadjusted OR (95% CI)	0.72 (0.40-1.30)		0.288
Acute Liver Failure, n (%)	195 (3.6)	1,677 (3.69)	0.89
Adjusted OR (95% CI)	0.99 (0.68-1.42)		0.956
Unadjusted OR (95% CI)	0.98 (0.68-1.38)		0.89
DIC, n (%)	15 (0.28)	164 (0.36)	0.6527
Adjusted OR (95% CI)	0.79 (0.25-2.48)		0.69
Unadjusted OR (95% CI)	0.76 (0.23-2.48)		0.654
Hepatic encephalopathy, n (%)	10 (0.18)	100 (0.22)	0.8134
Adjusted OR (95% CI)	1.185 (0.27-5.28)		0.819
Unadjusted OR (95% CI)	0.8395 (0.19-3.59)		0.814
Hepatorenal syndrome, n (%)	215 (3.97)	1,558 (3.43)	0.3635
Adjusted OR (95% CI)	1.077 (0.74-1.55)		0.689
Unadjusted OR (95% CI)	1.16 (0.38-1.61)		0.364
Ascites, n (%)	1,645 (30.41)	16,602 (36.54)	<0.001
Adjusted OR (95% CI)	0.72 (0.62-0.84)		<0.001
Unadjusted OR (95% CI)	0.76 (0.66-0.87)		<0.001
Portal HTN, n (%)	1,345 (24.86)	9,669 (21.28)	0.0092
Adjusted OR (95% CI)	1.12 (0.94-1.33)		0.2
Unadjusted OR (95% CI)	1.22 (1.05-1.42)		0.009
Total Charges ($)	109,108	85,406	<0.001
Adjusted coefficient (95% CI)	20458 (12050-28866)		<0.001
Unadjusted coefficient (95% CI)	23701 (15512-31890)		<0.001

## Discussion

Contrary to the current understanding regarding obesity and related HCC mortality, obesity was associated with reduced in-hospital mortality among patients admitted with a principal diagnosis of HCC in this study. However, obese patients with HCC were found to have indices of higher healthcare resource utilization measured in terms of prolonged length of stay and total hospitalization charges along with higher incidences of AKI during the hospital stay. Ascites, on the other hand, was found to be higher among non-obese patients. Other non-hepatic and hepatic complications such as the development of shock, sepsis, acute liver failure, disseminated intravascular coagulation, hepatic encephalopathy, hepatorenal syndrome, and portal hypertension were not found to be significantly different among obese and non-obese cohorts with HCC.

Several pathophysiological factors have been identified as potential contributors to the obesity-related risk of HCC. Obesity leads to a state of insulin resistance and compensatory hyperinsulinemia. Insulin leads to upregulation of hepatic growth hormone (GH), which leads to increased production of insulin-like growth factor-1 (IGF-1). IGF-1 is one of the potent activators of cellular proliferation via the protein kinase B signaling pathway, an inhibitor of apoptosis, and exerts a mitogenic effect via the activation of mitogen-activated protein kinases and expression of proto-oncogenes [11]. Hepatic lipid accumulation in obesity has been linked to lipotoxicity, which interferes with cellular signaling mechanisms and regulation of gene transcription, leading to the activation of oncogenic pathways. Lipotoxicity is also associated with free radical injury, which in itself leads to oxidative damage in genomic DNA, leading to carcinogenesis [12]. Other factors include endoplasmic reticulum stress secondary to the influx of fatty acid, which dysregulates ER proteostasis and leads to inflammation and tumorigenesis [13]. Low-grade chronic inflammation on obesity, oxidative stress, obesity accelerating the secretion of hepatokines that act as pro-inflammatory factors, individual susceptibility with genetic risk factors, disruption of epigenetic balance, etc. have also been postulated to play a role [14].

Although studies evaluating in-patient mortality among obese patients admitted with HCC are lacking, small-scale studies on obesity and survival outcomes among cancer patients have been reported. In a nationwide cancer registry database analysis evaluating the impact of obesity in post treatment overall survival in patients with HCC, overweight patients (25 kg/m^2^ ≤ BMI < 30 kg/m^2^) had the best prognosis, followed by normal weight (18.5 ≤ BMI < 25), obese (BMI ≥ 30 kg/m^2^) and underweight patients. It included stage 0, A, and B HCC patients who were treated with surgical resection, radiofrequency ablation, or transarterial chemoembolization. However, no significant difference in overall survival rates was seen in comparing overweight and normal-weight patients after propensity score matching. The differences in survival rates with poor prognosis among patients with low BMI were attributed to the impact of cachexia [15].

The effect of BMI on mortality was also reported on patients with colorectal carcinoma where overweight patients consistently had the best prognosis with the lowest risk of all causes and CRC-specific mortality. In contrast, extremes of weight at diagnosis were associated with elevated all-cause and CRC-related mortality. Extra muscle and fat mass in patients with overweight BMI was attributed to the ability to cope with the metabolic demands of tumor progression and treatment [16]. This is in contrast to the findings of a meta-analysis by Liu et al. where obese patients had a 1.84 times higher risk of HCC-related mortality. However, this effect could not be verified as an independent effect of obesity on HCC-related mortality as other factors such as etiology and severity of cirrhosis, HCC stage, and treatment were not adjusted for in individual studies [7]. Protective effect of obesity with in-patient mortality, as seen in our study, could be attributed to the obesity paradox with disease conditions where obesity contributes survival benefits, better nutritional status, lower production of tumor necrosis factor-alpha, preservation of vascular function, etc. [17-20].

Obese patients were found to have higher indices of healthcare utilization, i.e., higher length of stay and total hospitalization charges, along with higher rates of complications such as AKI in our study. This finding could be attributed to obese patients having higher CCI (i.e., ≥4 compared to the non-obese cohort). This translates to a higher comorbidity burden with underlying HCC and higher prospects of in-hospital complications, longer length of stay, and subsequent higher hospitalization charges. The findings of this study concur with the findings of an observational study on the impact of morbid obesity on the health outcomes of hospitalized patients where the length of stay was found to be significantly higher in the group with morbid obesity (BMI ≥ 40 kg/m^2^) than the non-obese group [21]. Similar findings were seen with a higher crude length of stay among patients with a BMI of 35 or above, those with a BMI of 30-34, and those with a BMI of 25-29 kg/m^2^ in a study by Zizza et al. [22]. Furthermore, hospital charges among obese patients were found to be 6.1% higher compared to non-obese patients after adjusting for diabetes status, age, sex, race, hospital admission type (such as emergency, urgent, etc.), and length of stay in a study that analyzed nearly eight million inpatient records from the NIS in 2005. Apart from obesity-related comorbidities, obesity in itself was independently shown to be associated with higher levels of healthcare resource utilization in the study [23]. Lastly, higher rates of AKI among obese patients could also be similarly attributed to the presence of disproportionately higher co-morbidities burden with underlying structural renal changes in obese patients [24].

This study has some inherent limitations. First and foremost, the study used the NIS database to obtain information on the study population. The NIS, being an administrative database, has a potential for misclassification of diseases using ICD-10 codes or missing codes. However, previous studies have used similar ICD-10 codes to obtain information regarding patients with obesity and HCC [25,26]. Second, obesity in our study was not stratified into different categories such as morbidly obese (BMI: >40 kg/m^2^) or obese (30 ≤ BMI ≤40 kg/m^2^), which have significant clinical implications with regard to outcomes of hospitalization, which could have confounded our results. Similarly, information regarding the staging and severity of HCC could not be obtained, which could have an impact on the outcomes of hospitalization.

## Conclusions

In conclusion, obesity is found to be associated with reduced in-hospital mortality among patients with HCC. However, obese patients with HCC were found to have higher healthcare resource utilization in terms of length of stay and total hospitalization charge along with the development of AKI. Clinicians should be mindful of the potential longer length of stay and associated complications such as AKI while managing obese patients with HCC. Contrary to commonly held notions, obesity and its relation with in-hospital mortality reported in this study warrant further explorative research.

## Appendices

**Table 3:** Study outcomes and appropriate ICD-10 codes used in the study ICD: International classification of diseases

Variable	ICD-10 code
Hepatocellular carcinoma	C22.0
Obesity	E66.XX
Ascites	R18.8, R18.0, K70.31, K70.11, K71.51
Hepatorenal syndrome	K76.7
Hepatic encephalopathy	K76.82, K72.91
Portal hypertension	K76.6
Acute liver failure	K72.00, K72.01
Disseminated intravascular coagulation	D65
Hepatitis B	B1910, B1911, B180, B181, B170, B178, B172, B169, B161
Alcoholism	F10.xxx
Hepatitis C	B1920, B1921, B180, B182, B1710, B1711
Acute kidney injury	N17.0, N17.1, N17.2, N17.8, N17.9
Sepsis	R65.10, R65.11, R65.20
Shock	R65.21, R57.1, R57.8, R57.9
